# New insights into malaria vector bionomics in Lao PDR: a nationwide entomology survey

**DOI:** 10.1186/s12936-020-03453-9

**Published:** 2020-11-09

**Authors:** Sébastien Marcombe, Santi Maithaviphet, Julie Bobichon, Nothasin Phommavan, Simone Nambanya, Vincent Corbel, Paul T. Brey

**Affiliations:** 1 Institut Pasteur du Laos, Ministry of Health, Vientiane, Lao PDR; 2 Center for Malariology, Parasitology and Entomology, Ministry of Health, Vientiane, Lao PDR; 3 Institut de Recherche Pour Le Développement (IRD), Maladies Infectieuses et Vecteurs, Ecologie, Génétique, Evolution et Contrôle (MIVEGEC, UM1-CNRS 5290-IRD 224), Montpellier, France

**Keywords:** Laos, Malaria, Primary and secondary vectors, Biting preferences, *Anopheles minimus*, *Anopheles maculatus*, *Anopheles dirus*, *Plasmodium infection*

## Abstract

**Background:**

In Laos, the malaria burden remains high despite a significant reduction of cases during the last decade. In the context of the disease elimination by 2030, a nationwide entomological survey was conducted to better understand the distribution, abundance and behaviour of major malaria vectors (*Anopheles* spp.) in the country.

**Methods:**

Mosquito collections were implemented in ten villages from ten provinces during the rainy and dry seasons of 2014 and 2015 by using human landing catch (HLC) and cow bait collection (CBC) methods. After morphological identification in the field, molecular identification of the sibling species of *Anopheles* mosquitoes from the Funestus, Leucosphyrus, and Maculatus groups were determined using PCR specific alleles. A screening of *Plasmodium falciparum* and *Plasmodium vivax* infections in the vectors was carried out by quantitative PCR assays.

**Results:**

A total of 14,146 adult mosquitoes representing 25 different *Anopheles* species were collected and morphologically identified. Molecular identification revealed the presence of 12 sibling species within the main primary vector groups, including *Anopheles maculatus*, *Anopheles rampae*, *Anopheles sawadwongporni*, *Anopheles pseudowillmori*, *Anopheles dravidicus*, *Anopheles minimus*, *Anopheles aconitus*, *Anopheles pampanai*, *Anopheles harrisoni*, *Anopheles dirus*, *Anopheles baimaii*, *Anopheles nemophilous*. *Anopheles maculatus* and *An. minimus* were predominant during both the dry and rainy seasons, but showed highly zoophilic preferences (Zoophilic index of 98% and 95%, respectively). Overall, 22% of the total malaria vectors were collected between 10:00 PM and 5:00 AM indoors when people are sleeping. Twenty-seven percent of primary and secondary vectors were collected outdoors before 10:00 PM or after 5:00 AM, times when people are usually awake and outdoors. Only two specimens were positive for *P. falciparum*, one *An. aconitus* from Phongsaly and one *An. minimus* from Vientiane Province

**Conclusions:**

The results indicate that people living in rural areas in Laos are constantly exposed to malaria vectors throughout the year and specifically outdoors. The use of LLINs/IRS remains important but innovative tools and new strategies are needed to address locally, the early and outdoor malaria transmission. Lack of expertise in general entomological methods may further exacerbate the situation.

## Background

Malaria is the deadliest vector-borne disease worldwide with an estimated 228 million cases, and 405,000 deaths in 2018, mostly in the African Region (93%), followed by the South-East Asia Region (3.4%) [1]. Despite a continued decline of malaria cases (by 74%) and deaths (by 94%) in the last decade in the Greater Mekong Subregion (GMS), malaria remains a major public health problem impacting on the health and lives of a large proportion of people particularly in remote areas and concentrated along international borders [2–4]. In Laos, malaria transmission is heterogeneous, with more intense transmission in forested areas particularly in the southern part of the country [5], where more than 207,000 cases were reported in 2018. The country has implemented a nation-wide malaria control programme since 1992, and the current strategies emphasize the promotion of long-lasting insecticide-treated bed nets (LLINs), early diagnosis by microscopic examination and rapid diagnostic tests, and prompt treatment with artemisinin-based combination therapy (ACT). Even if the burden of malaria remains high, there have been a large decrease in the number of cases during the past decades and following these encouraging results, the Laos Ministry of Health has planned the elimination of the diseases by 2030 [6]. The regional strategy for malaria elimination aims at eliminating malaria foci in all GMS countries, maintaining a malaria-free status, and preventing reintroduction. The strategy also includes urgent action to eliminate *Plasmodium falciparum* malaria by 2025 to contain the spread of multi-drug resistance in the GMS and in the southern part of Laos, specifically [1, 3, 7].

In the context of elimination, entomological aspects of malaria transmission are crucial in order to devise, and implement effective, scalable and locally-adapted vector control interventions [4]. The disease control efforts globally in Laos and in the GMS have depended largely on the use of pyrethroid-based insecticide-treated nets and indoor residual spraying strategy (IRS). These interventions have greatly contributed to significantly reduce the burden of malaria in the GMS where the disease elimination is a priority [1]. The efficacy of any vector control interventions is strongly influenced by the ecology and behaviour of malaria vectors [8]. To guide the choice of control strategy to apply in the field, it is highly desirable to have recent data on (i) the vector composition, diversity and abundance including sibling species and (ii) the spatial and temporal distribution patterns of potential vectors and (iii) presence/absence of any resistance to public health insecticides. A previous study was implemented at the same locations presented later in this paper, on the susceptibility of *Anopheles* species, including malaria vectors (*Anopheles dirus*, *Anopheles maculatus*, and *Anopheles minimus*) and the results showed that pyrethroid resistance in the main malaria vectors is absent in Laos [9]. There are still some knowledge gaps however, in the understanding of malaria vectors bionomic in the region, as well as in their role in *Plasmodium* spp. transmission. In Laos, 170 mosquito taxa have been officially reported, of which 42 *Anopheles* species and *Anopheles dirus*, *An. maculatus* and, *An. minimus* are considered as the major malaria vectors [10–16]. Other potential vectors, such as *Anopheles aconitus*, *Anopheles barbirostris*, *Anopheles nivipes* and, *Anopheles philippinensis* are present [10], but little is known about their vectorial capacity and competence for *Plasmodium* transmission.

The present study was conducted in the framework of a nation-wide entomology surveillance (Malaria Vector Project (MALVEC) project, 2013–2016) aiming at filling knowledge gaps in malaria vector bionomic and insecticide resistance in Laos. The objective of this paper is to describe the distribution, seasonal abundance and, biting behaviour of malaria vectors throughout a North–South transect in ten provinces of the country to guide policy making for vector control and malaria elimination.

## Methods

### Study areas and mosquito collections

**Fig. 1:**
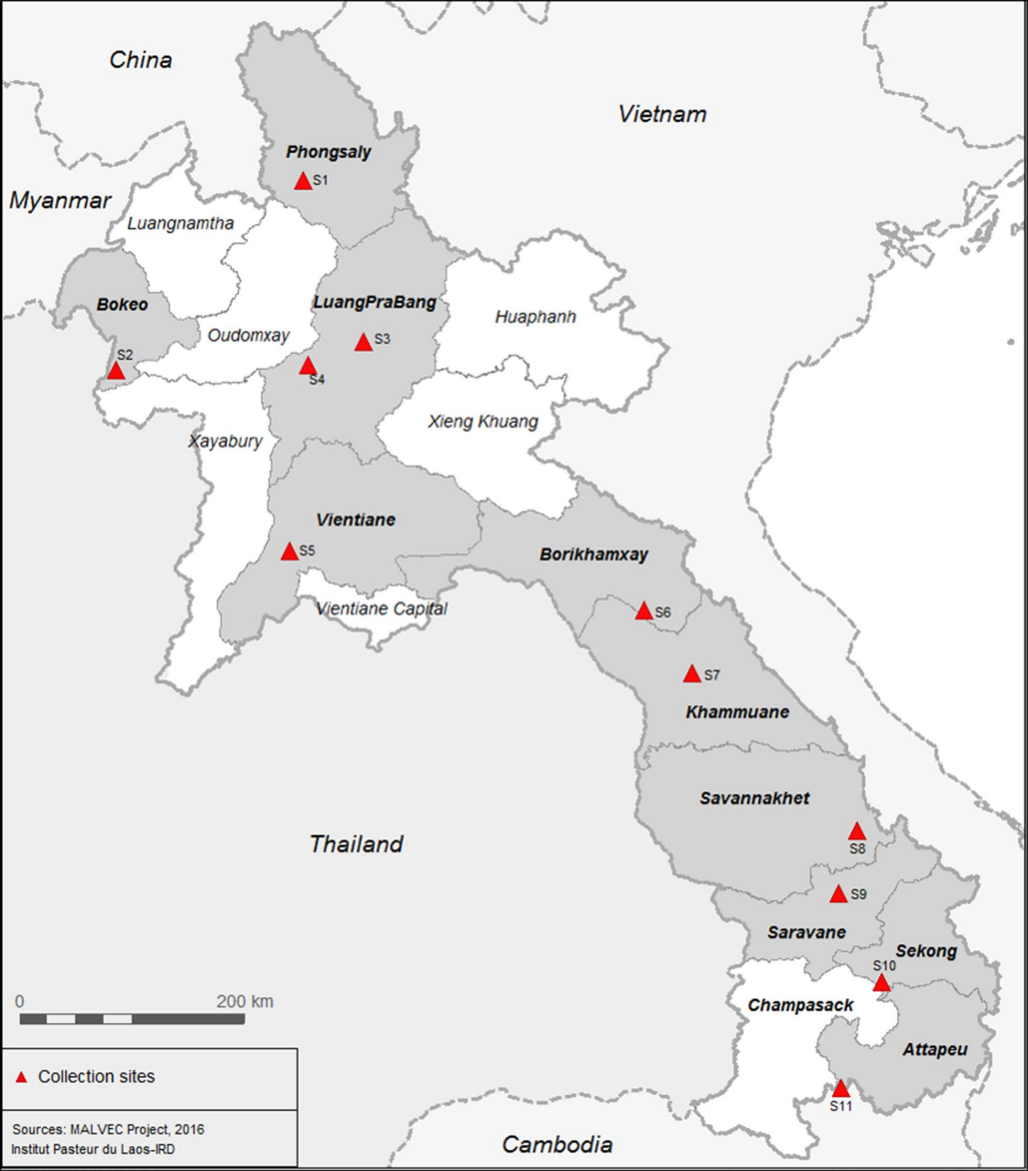
Location of the collection sites in Laos [9]

Entomology surveys were implemented in ten villages from ten provinces in Laos where malaria transmission is known to occur (Table 1 and Fig. 1) [9]. Mosquito collections were conducted during both the rainy (June to October) and dry (January to May) seasons in 2014 and 2015. Human landing catch (HLC) and cow bait collection (CBC) were used to catch both zoophagic and anthropophagic mosquitoes. Mosquitoes were collected by volunteers recruited in each village from 18:00 to 06:00 during four consecutive nights. In each village, four households were used for HLC (inside and outside). Villages were divided into four zones from a central axis to select at random one house per quadrant. The four houses were located at least 30 m from each other. In each of the selected four houses, a village inhabitant collected mosquitoes inside the house and another outside the house. A rotation of collectors between the houses was implemented and coordinated by the supervisors to mitigate potential collector bias. Using glass tubes, collectors caught mosquitoes from their exposed legs. Animal bait collections were carried out by placing a 25 m long and 1.5 m height cotton mosquito net around it (cow or buffalo). The net was suspended 30 cm above the ground level to allow mosquitoes to access the animal. The catch site used for CBC was at least 100 m away from any HLC catch site to avoid potential interference between the two methods. Adult mosquitoes landing on the net were collected by one collector for 15 min each hour between 18:00 and 06:00. For both HLC and CBC, mosquitoes were collected and stored individually in glass tubes and were provided with 10% sugar solution until morphological identification. The number of mosquitoes collected every hour was recorded by the supervisors.

**Table 1:** Mosquito collection sites in Laos [9]

Site number	Province	District	Village	Latitude	Longitude
S1	Phongsaly	Bountai	Boulykao	21.33778	102.08247
S2	Bokeo	Paktha	Hadsa	19.92268	100.58148
S3	Luang Prabang	Pakseng	Sopjak	20.13477	102.55834
S4	Luang Prabang	Chomphet	Na	19.96715	102.11792
S5	Vientiane Pro	Feuang	Na-ang	18.55996	101.97389
S6	Borlikhamxay	Khamkeut	Phameung	18.11425	104.80229
S7	Khammouane	Gnommalath	Koutphadang	17.63663	105.17795
S8	Savannakhet	Nong	Sadi	16.43901	106.50284
S9	Saravane	Toomlarn	Katao	15.95187	106.35285
S10	Sekong	Lamam	Lavynoy	15.27291	106.69748
S11	Attapeu	Sanamxay	Hadoudomxay	14.45668	106.36727

### Morphological identification

The next morning following collections, mosquitoes from both HLC and CBC were morphologically identified to genus (*Aedes*, *Anopheles*, *Armigeres* and *Culex*) and to species or group/complex in field laboratory, using microscopes and appropriate identification keys for Southeast Asian anophelines [17]. Identification was carried out on site by qualified entomologists from the Institut Pasteur du Laos (IPL) and the Center for Malariology, Parasitology, and Epidemiology (CMPE). After identification, mosquitoes of the same species were stored in RNAlater^®^ or in silica gel in labelled 1.5 ml tube and stored at – 20 ºC. Mosquitoes were then brought back to the Entomology laboratory of IPL in Vientiane for subsequent laboratory analysis.

### Sibling species identification, allele specific-PCR

All field caught *Anopheles* female mosquitoes belonging to primary vectors (*An*. *minimus* and *An. dirus* complexes and *An. maculatus* group) were cut in two parts to separate the head and thorax from the abdomen. Head and thorax were subjected to DNA extraction using a commercial extraction kit (Nucleospin virus, MACHERY-NAGEL GmbH & Co. KG) according to the manufacturer’s instruction. DNA was used for molecular detection of sibling species within the Dirus complex, Maculatus group, and Minimus complex assemblages by using an allele-specific multiplex assay (AS-PCR) examining the ITS-2 region of the DNA [18–20].

The following modifications were added in the protocols: the amplification was carried out in a 25 μL reaction mixture containing 1 unit of TfiDNA polymerase (Invitrogen), 200 μM of dNTPs, 2 mM of MgCl_2_, 400 nM of each primer, and 4 μL of DNA template at working concentration. For the Minimus complex, after an initial denaturation step at 94 °C for 2 min, 40 cycles were programmed as follows: 94 °C for 30 s, 45 °C for 30 s, 72 °C for 40 s, and a final extension step at 72 °C for 5 min. For the Maculatus group, after an initial denaturation step at 94 °C for 5 min, 35 cycles were programmed as follows: 94 °C for 1 min, 55 °C for 30 s, 72 °C for 30 s, and a final extension step at 72 °C for 10 min. For the Dirus complex, after an initial denaturation step at 94 °C for 5 min, 35 cycles were programmed as follows: 94 °C for 15 s, 55 °C for 15 s, 72 °C for 30 s, and a final extension step at 72 °C for 10 min. The PCR products were subjected to electrophoresis on 2% agarose gel with ethidium bromide at 100 V for 30 min and bands were visualized by UV transillumination.

### Plasmodium detection, qPCR assays

DNA extracts of pools of three specimens of sibling species were screened for *P. falciparum* and *Plasmodium vivax*, and *Plasmodium knowlesi* and were performed with a MiniOpticon real time PCR system (Bio-rad, CFX Manager 3.0 software). For the screening of *P. falciparum* and *P. vivax*, the sequences of the primers were provided by NCGM (National Center for Global Health and Medicine, Japan) and are described in Canier et al*.* [21], but with a modification for the RTPCR Screening2_R primer (TTGCACCCCAATARCTCATTT). For *P. knowlesi*, the primers were developed during the study (PkF: GAG TT A TTG GGG TGC AAC TGT C and PkR (CTG TAT ATC CTC CAC ATAACC AAA TG). Reactions were conducted using 9.5 μL of SYBR Green Super Mix (Bio-rad), 0.25 μL of each primer F and R and 2 μL of DNA template for a total reaction volume of 20 μL. The thermocycling protocol was 95 °C for 3 min followed by 40 amplification cycles at 95 °C for 10 s, 60 °C for 30 s and dissociation. Positive pools were confirmed with the melt curve analysis. Positive controls were provided by NCGM from mosquitoes artificially-infected with *P. falciparum* and *P. vivax*. These infected mosquitoes were extracted in the same condition as the samples used in this study. All positive samples were confirmed for *Plasmodium* species by sequencing (NCGM, Japan).

### Data analysis

The human biting rates and the cow biting rates were calculated as follows:

1. HBR = No. mosquitoes collected on human volunteers/No. of human-nights,
2. CBR = No. mosquitoes collected on cow bait/No. of cow-nights,
  The human biting rates indoors and outdoors were calculated as follows:
3. HBR indoors = No. mosquitoes collected on human volunteers indoors/No. of human-nights indoors,
4. HBR outdoors = No. mosquitoes collected on human volunteer outdoors/No. of human-nights outdoors.
  The anthropophagic and zoophagic indices were calculated as follows and these indicators (ZI and AI) are used as a proxy to estimate the “host-seeking” preferences based on the relative number of mosquitoes collected on different host:
5. Anthropophagic Index (AI) = HBR/(HBR + CBR).
6. Zoophagic Index (ZI) = CBR/(HBR + CBR).
  The endophagic and exophagic indices were calculated as follows:
7. Endophagic index (EnI) = HBR indoors/(HBR indoors + HBR outdoors).
8. Exophagic index (ExI) = HBR outdoors/(HBR indoors + HBR outdoors).

## Results

### Anopheles diversity, abundance and host-seeking preference

Table 1 shows the abundance of *Anopheles* collected according to the host (cow or human). A total of 14,146 adult mosquitoes representing 25 different *Anopheles* species were collected and morphologically identified in the field. Overall, only five species (i.e. *An. minimus *sensu lato (s.l.), *An. nivipes *s.l*.*, *An. maculatus *s.l., *An. aconitus* and *Anopheles vagus*,) accounted for 75% of the total number of mosquitoes collected. Four of them are considered as either primary or secondary malaria vectors in Laos [16]. Among the primary vectors, *An. minimus *s.l. was the most abundant species collected on both cows and humans (n = 1875 and 882, respectively, 19.4% of the total) followed by *An. maculatus *s.l. (n = 1658 and 221, respectively, 13.3%). The other primary vector *Anopheles dirus *s.l. constituted only 0.3% (n = 43) of the total number of mosquitoes collected (n = 33 on human and n = 10 on cows). *Anopheles maculatus *s.l. and *An. minimus *s.l. were found in all provinces, whereas *An. dirus* was only found in six provinces (Additional file 1: Table S1). The most abundant secondary malaria vector species was *An. nivipes *s.l., which constituted 19.1% of all mosquitoes collected in nine provinces. The other secondary vectors, *An. aconitus*, *An. philippinensis* and *An. barbirostris *s.l. represented 10.6%, 4.8% and 1.9%, respectively. *Anopheles vagus*, considered as a non-malaria vector in Laos, represented more than 12% of the total mosquitoes collected. The highest number of *Anopheles* collected was in Vientiane province (n = 3883 and 27% of the total). In the southern Laos, the province with the highest *Anopheles* abundance was Attapeu, bordering Cambodia, with 1971 mosquitoes collected, hence representing 14% of the total. In the northern Laos, Phongsaly province, bordering China and Vietnam, exhibited a high density of *Anopheles* mosquitoes (n = 1800, 13% of the total, Additional file 1: Table S1). About 78% of mosquitoes (n = 11,155) were collected on cows, whereas 21% were caught on humans (n = 2991). All species, except for *An. dirus *s.l.*,* were found biting on both baits showing that the two methods, HLC and CBC, gave similar results with regards to malaria vectors composition.

### Sibling species identification

**Fig. 2:**
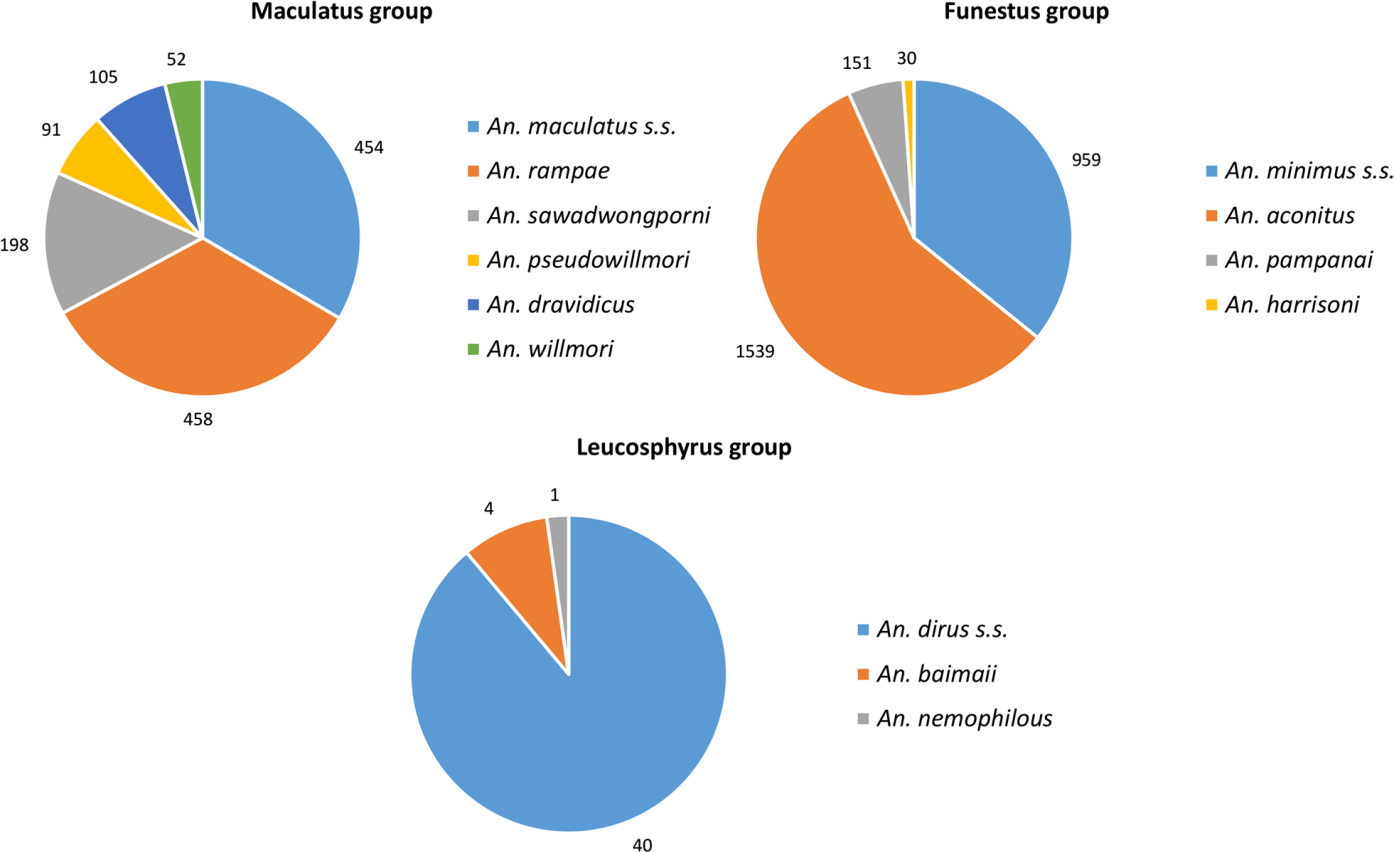
Number of Sibling species of *Anopheles* mosquitoes belonging to the Maculatus, Funestus and Leucosphyrus groups determined by PCR and sequencing methods

A total of 4247 *Anopheles* mosquitoes belonging to the Maculatus, Funestus and Leucosphyrus groups collected in the field, were identified in the laboratory using conventional PCR or sequencing methods (Fig. 2). A total of 1387 *Anopheles* mosquitoes belonging to the Maculatus group were identified (Additional file 2: Table S2). All the species identified and listed under Maculatus Group are sibling species that belong to the Maculatus complex. The sibling species detected were *An. maculatus *sensu stricto (s.s.) (n = 454), *Anopheles rampae* (n = 458), *Anopheles sawadwongporni* (n = 198), *Anopheles pseudowillmori* (n = 91), and *Anopheles dravidicus* (n = 105). In addition, 83 specimens could not be identified probably due to problems occurring during DNA extraction or transport between the field and the laboratory. A total of 2825 *Anopheles* mosquitoes belonging to the Funestus group were also identified (Additional file 2: Table S2). Minimus complex is one of the complexes under the Funestus group. The two species identified in this study, *An. minimus *s.s. and *An. harrisoni* are members of the Minimus Complex [22]. The Minimus Complex includes three species, *An. minimus* (formerly minimus species A) [23, 24], *An. harrisoni* (formerly minimus species C) [23, 25] and *Anopheles yaeyamaensis* (formerly minimus species E) [26]. Adult females of *An. aconitus* are morphologically very similar to *An. minimus* and sometimes difficult to distinguish under microscope. To avoid any misidentification, the *An. aconitus* collected were also identified with molecular tools. PCR identifications showed that 959 samples were *An. minimus *s.s. (sp. A), 1539 were *An. aconitus*, 151 were *Anopheles pampanai*, 30 were *An. harrisoni*, 32 were negatives and, 150 were misidentified in the field as belonging to the Funestus group. Leucosphyrus group has three sub-groups, and Leucosphyrus sub-group is one. Under the Leucosphyrus sub-group there are two species complexes, Dirus and Leucosphyrus. Under the Dirus and the Leucosphyrus complexes, seven and four sibling species, respectively, were identified. The three species identified in this study, *An. dirus *s.s, *An. baimaii* and *Anopheles nemophilus* belong to the Dirus Complex [27]. A total of 35 *Anopheles* mosquitoes belonging to the Leucosphyrus group were identified (Additional file 2: Table S2) and PCR identifications showed that 40 samples were *An. dirus *s.s. (sp. A), 4 were *An. baimaii*, 1 was *An. nemophilous* and 6 were misidentified in the field as belonging to this group.

**Fig. 3:**
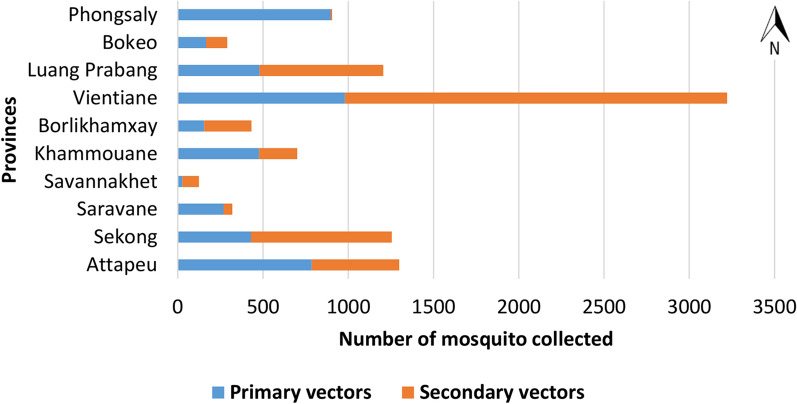
Total number of primary and secondary malaria vectors collected in Laos, in 2014 and 2015 (132 nights of collection). Species were identified by morphological methods

The proportions of primary and secondary vectors collected per province (from north to south) are shown in Fig. 3. Primary and secondary vectors were found in all ten provinces, but secondary vectors were slightly more abundant than primary vectors (n = 5083 vs. n = 4669). Vientiane province had the highest number of primary (n = 981) and secondary vectors (n = 2241) compared to the other provinces, followed by Attapeu and Sekong (n = 1299, and 1255 specimens, respectively). In Phongsaly province, more than 99% of the mosquitoes collected were primary vectors. Relatively high proportions of primary vectors were also collected in the southern provinces of Saravane, Khammouane, Bokeo and Attapeu provinces. In Luang Prabang, Vientiane, Borlikhamxay and Sekong, secondary vectors were more abundant.

**Fig. 4:**
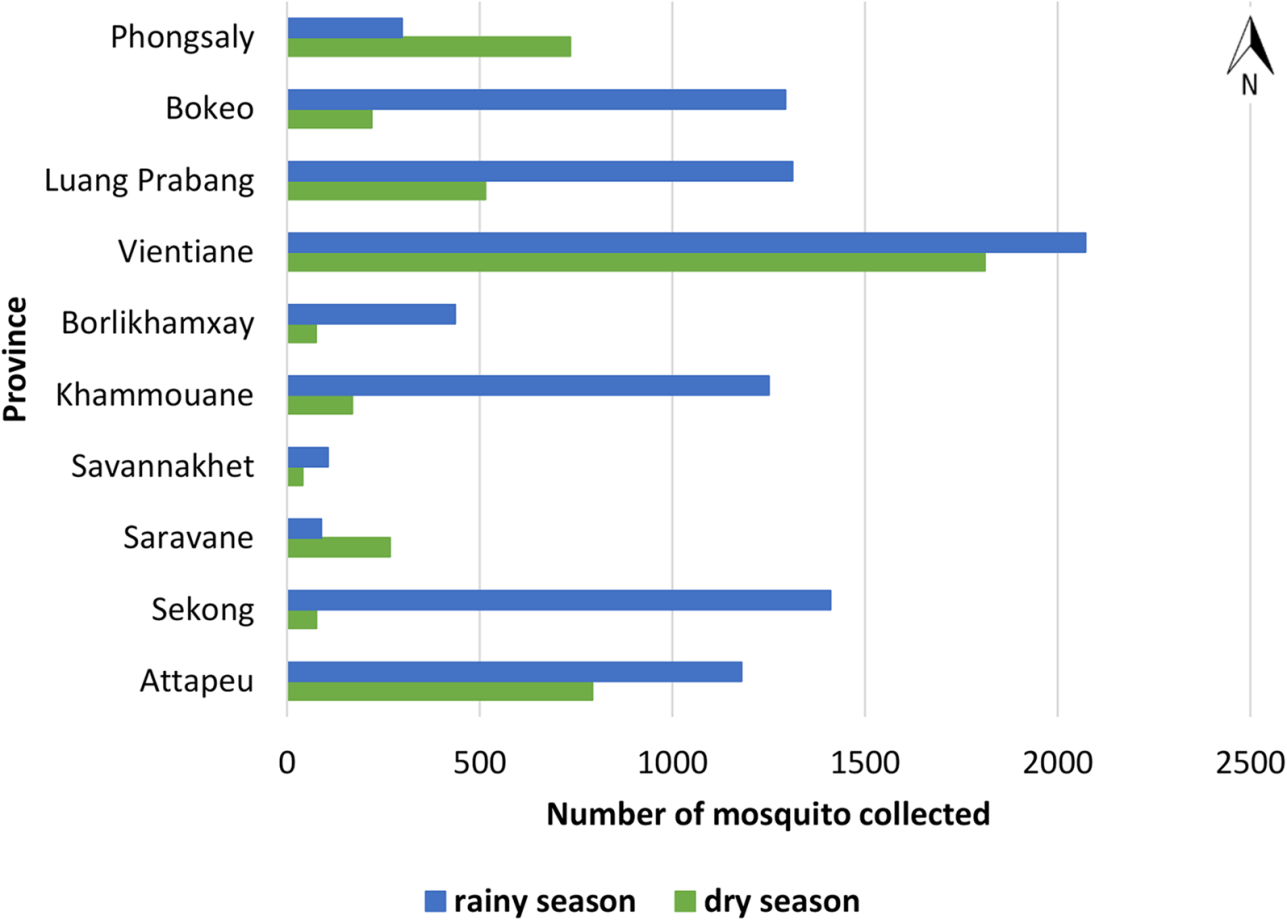
Abundance of *Anopheles* spp. mosquitoes collected in Laos according to season in 2014 and 2015 (132 nights of collection)

Figure 4 shows the number of *Anopheles* spp. collected during the dry and rainy seasons in 2014–2015 (132 nights of collection). More mosquitoes were collected during the rainy season (n = 9472, representing 68% of the total) than the dry season (n = 4423, 32%). On the contrary, more mosquitoes were collected during the dry season in Phongsaly and Saravane provinces.

### Host-seeking preference and biting behaviour

**Fig. 5:**
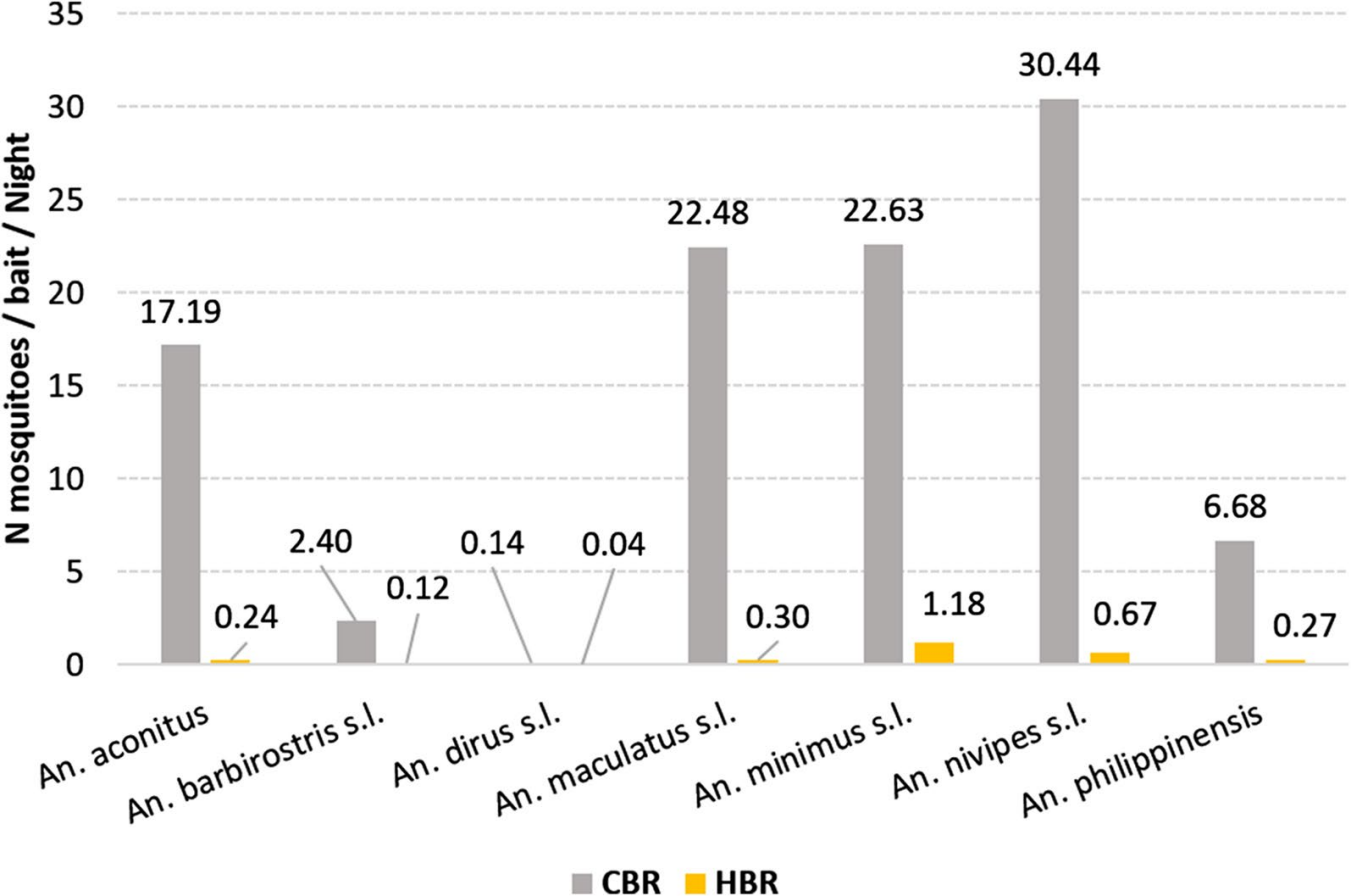
Host seeking preference of primary and secondary vectors in Laos in 2014 and 2015. cow biting rates (CBR) and human biting rates (HBR)

Figure 5 shows the host biting preference of the primary and secondary malaria vectors as measured by the CBR (A) and HBR (B). Data from dry and rainy seasons of 2014 and 2015 were pooled. For all species, including malaria vectors, CBRs were much higher than HBRS (i.e. from 3.5 to 72 fold higher according to the species). The highest CBRs were reported for *An. nivipes *s.l., *An. maculatus *s.l. and *An. minimus *s.l. (CBR = 30.44, 22.48 and 22.63, respectively), whereas the lowest CBRs were observed for *An. dirus *s.l. (CBR = 0.14). The HBRs varied between 0.04 (*An. dirus *s.l.) and 1.18 (*An. minimus* s.l.).

**Fig. 6:**
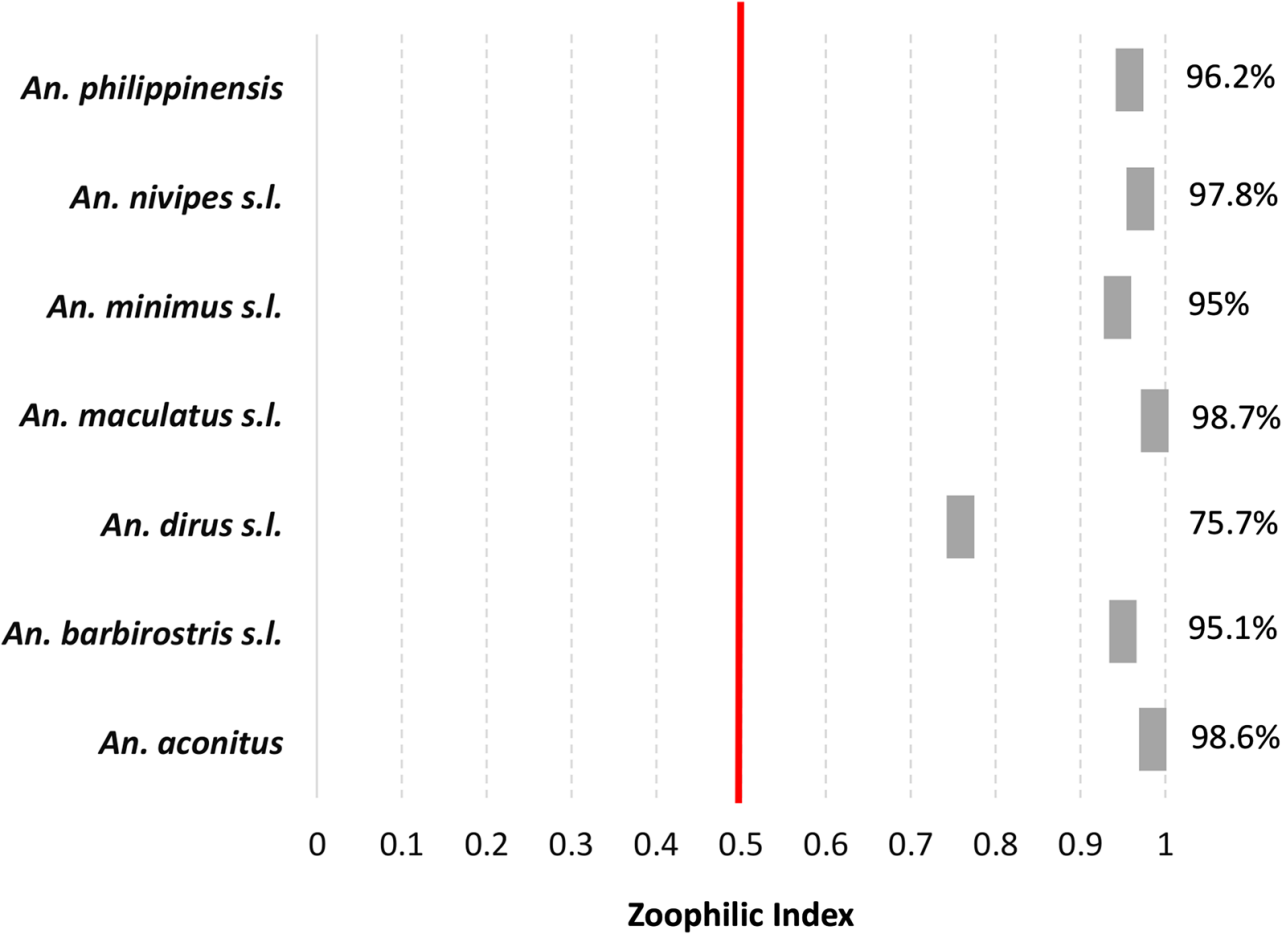
Zoophagic index of the *Anopheles* spp. collected in Laos in 2014 and 2015. ZI calculated as: CBR/(CBR + HBR)

Overall, *An. dirus* was the most anthropophagic species collected with a zoophagic index (ZI) of 75% (Fig. 6). The two other primary vectors, *An. maculatus *s.l. and *An. minimus *s.l. showed higher zoophagic indices (ZI = 98% and 95%, respectively). All secondary vectors were mostly zoophagic (> 95%).

**Fig. 7:**
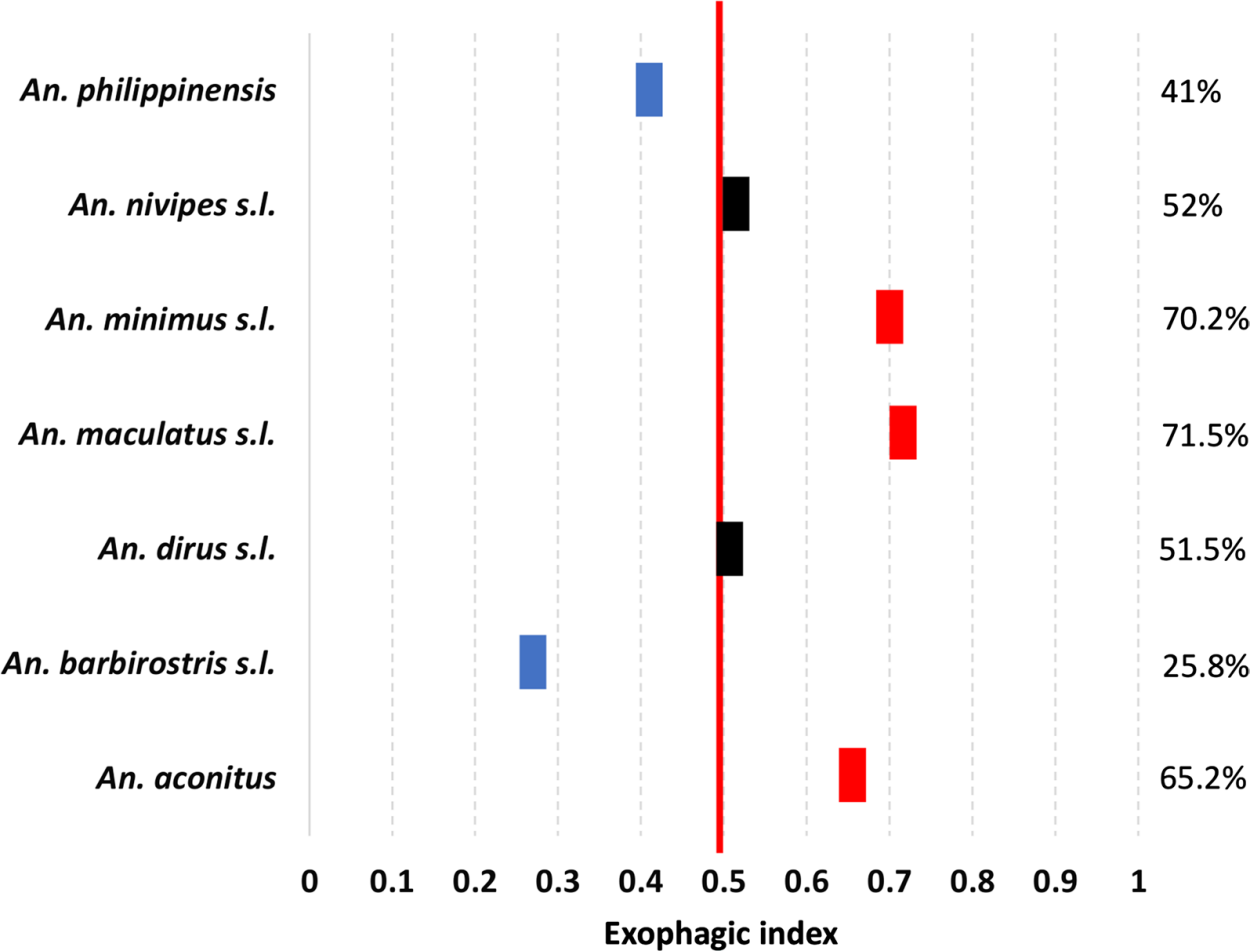
Exophagic index of the *Anopheles* sp. collected in Laos in 2014 and 2015. Exophagic index calculated as: HBR outdoors/(HBR outdoors + HBRindoors)

Among mosquitoes collected on humans, 41% were collected indoors and 59% outdoors (n = 1232 and 1759, respectively). The primary vectors *An. maculatus *s.l. and *An. minimus *s.l. were more active outdoors with exophagic indexes of 70.2% and 71.5% (Fig. 7), respectively (HBR of 0.3 and 1.18, and CBR of 22.5 and 22.6, respectively). *Anopheles dirus *s.l. and *An. nivipes *s.l. were collected almost equally outdoors and indoors (51.5% and 52%, respectively; HBR of 0.04 and 0.67, and CBR of 0.14 and 30.4, respectively). The most endophagic species were the secondary vectors *An. barbirostris* and *An. philippinensis* with endophagic indexes of 74% and 59%, respectively (HBR of 0.12 and 0.27 and CBR of 2.4 and 6.7, respectively).

**Fig. 8:**
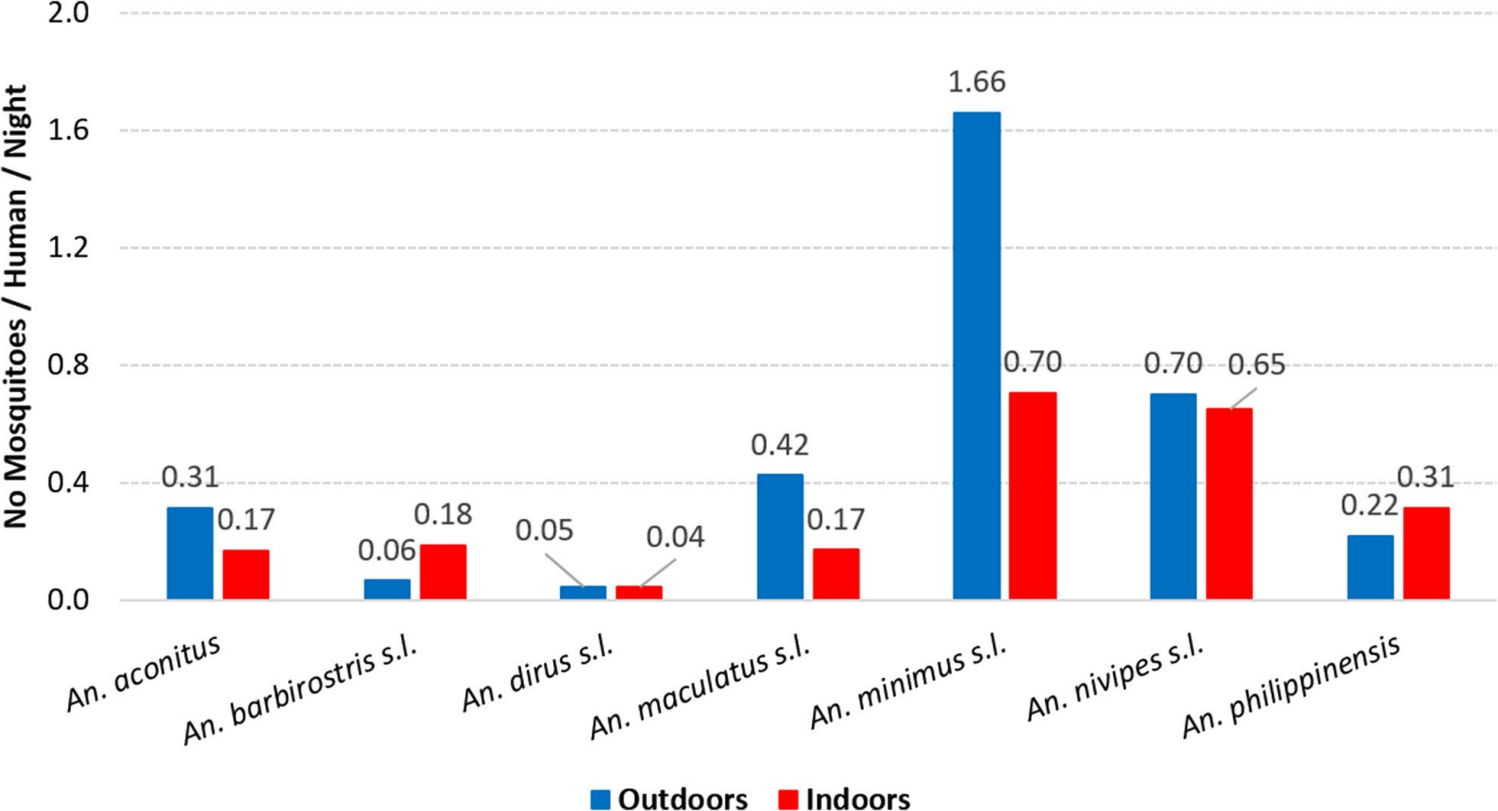
Outdoors and indoors biting rates of the *Anopheles* mosquitoes collected in Laos in 2014 and 2015

The human biting rates indoors varied from 0.04 to 0.7 and the HBRs outdoors from 0.05 to 1.66 (Fig. 8). The HBRs of *An. minimus *s.l. were the highest both indoors and outdoors (HBR = 0.7 and 1.66, respectively) followed by *An. nivipes *s.l. (HBR = 0.65 and 0.7, respectively). In contrast, *An. dirus *s.l. had the lowest HBRs for both indoors and outdoors (HBRI = 0.04 and HBRO = 0.05). The indoor and outdoor HBR of *An. maculatus *s.l. was 0.17 and 0.42, respectively.

### Hourly biting time

**Fig. 9:**
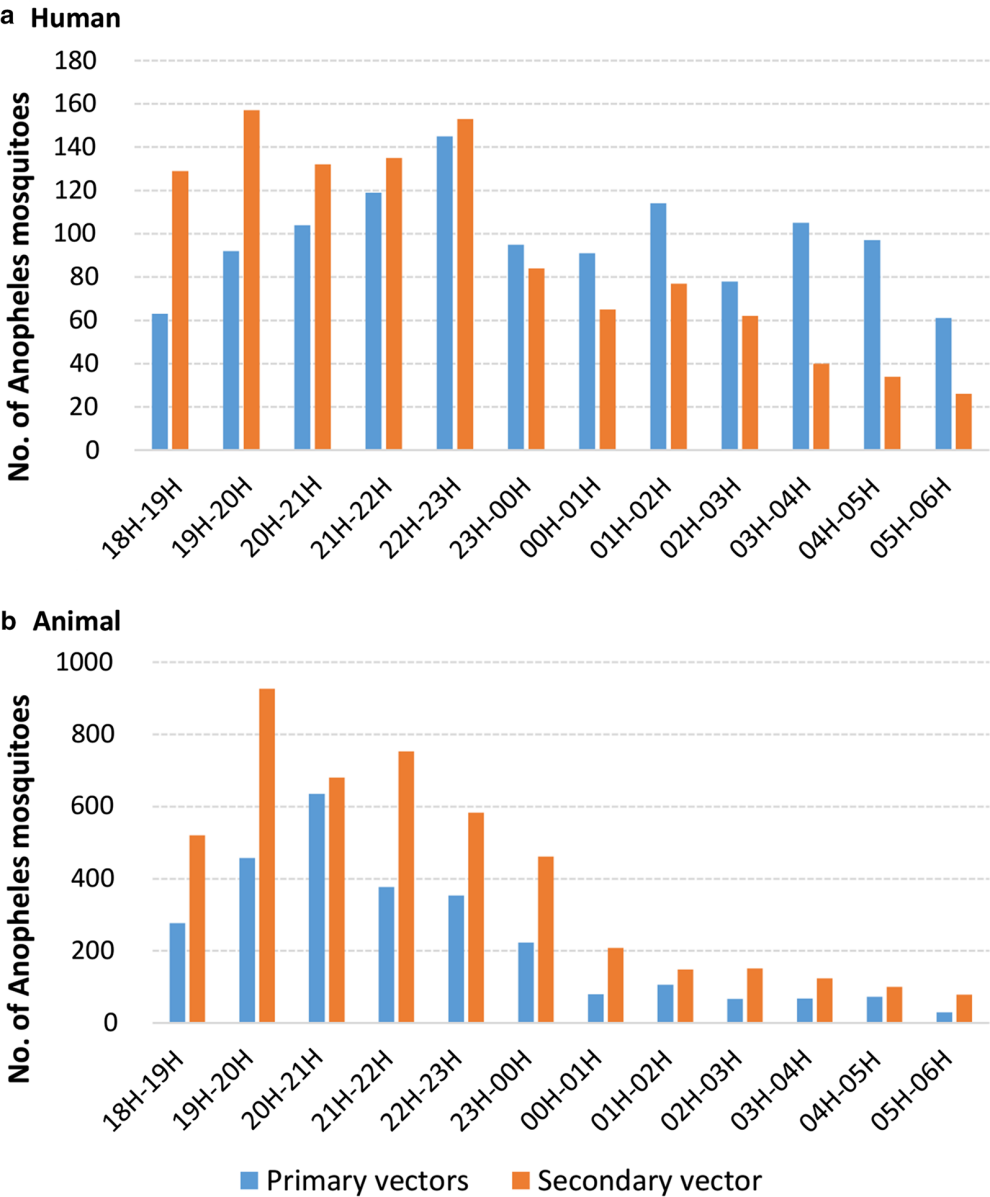
Biting times on human (**a**) and on animal (**b**) of the malaria vectors collected between 6:00 PM and 6:00 AM in Laos in 2014 and 2015 (132 nights of collection)

Primary and secondary vectors had different peak of activities during the night depending on whether they were collected on human or cow bait (Fig. 9). The primary vectors were biting humans throughout the night, with the highest abundance in the early evening and reaching a peak between 10:00 and 11:00 PM (Fig. 9). The secondary vectors biting humans were more active before midnight and their activity then decreased until 6 AM. The activity pattern of primary and secondary vectors biting cows were similar with a high abundance before midnight (> 500 mosquitoes collected per hour) following by a decreased activity until 6:00 AM (Fig. 9).

**Fig. 10:**
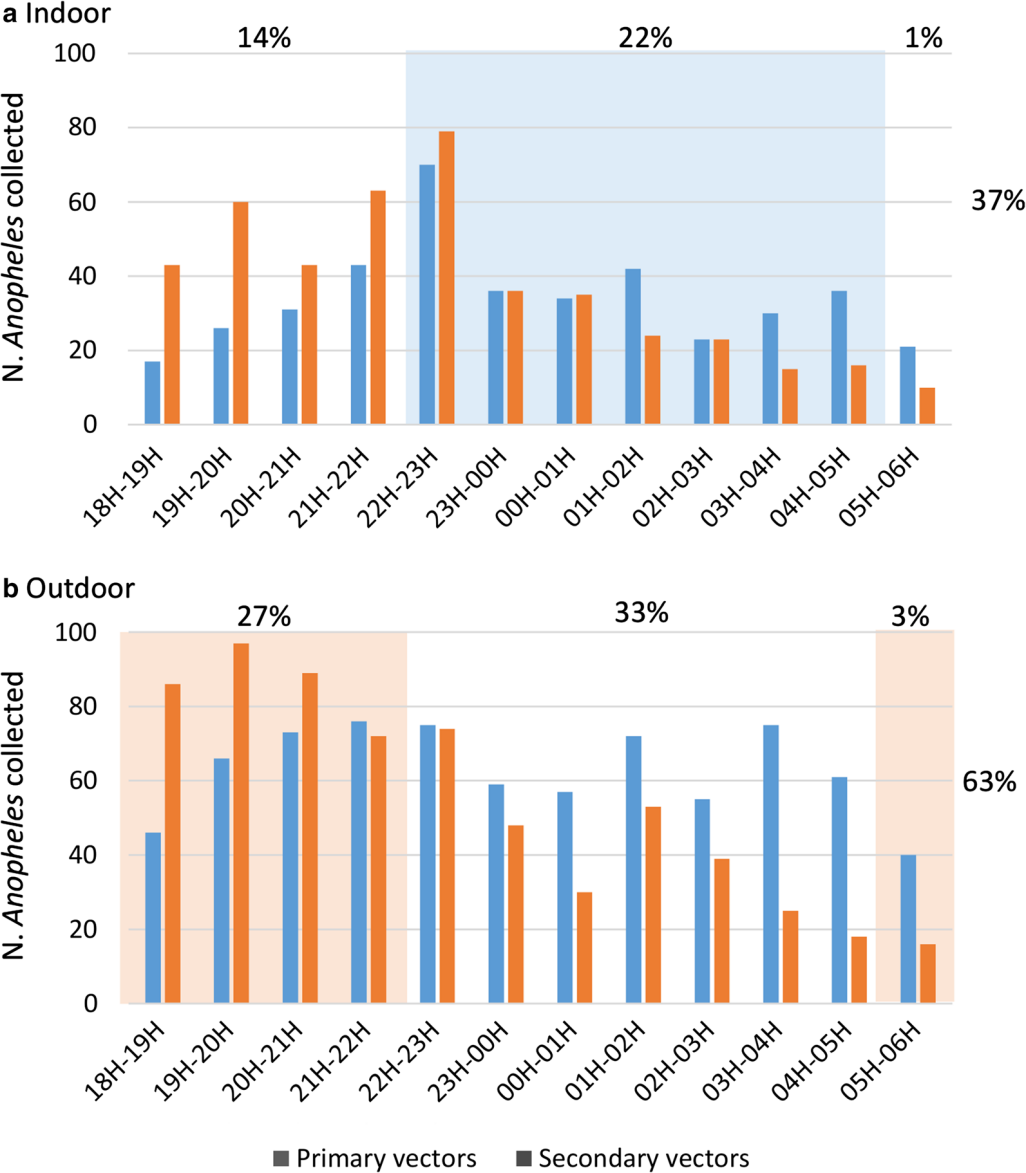
Biting times of the malaria vectors on humans indoors (**a**) and outdoors (**b**) in Laos during 2014 and 2015 collections. The blue area represents the hours when people are potentially protected by LLINS and the red area the hours when people are not protected outside

The numbers of mosquitoes collected hourly on humans, both inside and outside houses, are shown in Fig. 10. Primary and secondary vectors were active indoors and outdoors throughout the night. More specifically, 22% of the malaria vectors (both primary and secondary) were collected indoors on humans between 10:00 PM and 5:00 AM when the people were supposedly sleeping inside under a bed net. Twenty-seven percent of primary and secondary vectors were collected outdoors between 6:00 PM and 10:00 PM, when people are still active outside. Also, 14% of the vector were collected indoors when people may not be inside LLINs and are vulnerable to biting by primary vector species. The Additional file 3: Table S3 shows the biting time of the vectors in details.

### *Plasmodium* infection

A total of 4192 mosquitoes from the Maculatus, Minimus and Dirus groups and complexes, secondary vectors and possible vectors were screened for *Plasmodium* sp. detection. Among them only one *An. aconitus* from Phongsaly province and one *An. minimus *s.s. from Vientiane province were positive for *P. falciparum* (sporozoite rate of 0.04% [2/4192], Additional file 4: Table S4). These results were confirmed by sequencing. Unfortunately, it was not possible to determine if these specimens were collected on animal or human, and indoors or outdoors, because the mosquitoes were pooled in the field for the conduct of insecticide resistance bioassays [9].

## Discussion

This nationwide entomological survey documents the bionomics of malaria vectors in ten sites of Laos where malaria elimination is planned for 2030. This is the largest entomological study implemented in the country since the pioneer work of the MALVECASIA project in the 2000’s [28, 29]. These data are timely and of great importance to guide decision-making in vector control in the country. Indeed, accurate information on vector composition, bionomic and distribution (both spatial and temporal), are keys to design and implement scalable and locally-adapted vector control interventions.

Results showed a great diversity of *Anopheles* species in the study areas with 25 different species/complexes morphologically identified (Additional file 1: Table S1). All the *Anopheles* complex species collected were already described in Laos [10–12, 30–32], but 13 *Anopheles* spp. from three different groups (i.e. Funestus, Leucosphyrus and, Maculatus) could be identified for the first time for some species, using molecular tools. These findings provide additional information to the checklist of the 42 *Anopheles* species of Laos recently updated by Motoki et al. [30]. Moreover, this provides us important data on the relative proportions of primary versus secondary vectors within the three groups mentioned above. For example, the three primary vector species, identified by qPCR, *An. dirus *s.s., *An. maculatus *s.s. and, *An. minimus *s.s. represented 89, 36 and, 33% of the total mosquitoes of the Dirus and Minimus complexes and Maculatus group, respectively. In the Maculatus group, *An. rampae* represented 34% of the total but this zoophilic species is not a malaria vector [33]. In contrast, within the same group *An. sawadwongporni* represented 14% of the total and is considered as a very efficient vector in Thailand [34]. Within the Minimus complex, *An. aconitus* accounted for 57% of the total and represented more than 10% of the total number of the mosquitoes collected. It should be noted that recently, Taai et al*.* [22] indicated that this species is morphologically closely resemble *An. minimus* but is not part of the Minimus complex, and can be listed as part of the Funestus Group. This species is highly zoophilic and exophagic [35, 36], and Manh et al. [37] showed it was to some extent, responsible to maintain transmission in rural communities, and deforested areas in north-central Vietnam. The abundance of other secondary malaria vectors was relatively high, such as *An. nivipes* (19% of all *Anopheles* spp. collected), *An. philippinensis* (4.8%) and, to a lesser extent *An. barbirostris* (1.9%). All these species are mostly zoophilic, but they can also bite humans. Several studies implemented in Laos [12, 31, 38] showed that *An. philippinensis* and *An. nivipes* are able to bite both human and animals and the authors suspected them to be responsible for malaria transmission in paddy field areas in Khammouane province. Indeed, both of these species were previously found infected by *P. falciparum* or *P. vivax* in Laos and in other GMS countries [12, 17, 39]. Clearly more work has to be done to determine the behaviour and ecology of secondary vectors and their role in transmission (Table 2).

**Table 2:** Abundance and diversity of morphologically identified *Anopheles* mosquitoes collected in Laos in 2014 and 2015

Anopheline taxa	CBC		HLC		Total	
	N	%	N	%	N	%
*An. minimus* s.l.^a^	1865	13.2	882	6.2	2747	19.4
*An. nivipes* s.l.^b^	2207	15.6	501	3.5	2708	19.1
*An. maculatus* group^a^	1658	11.7	222	1.6	1880	13.3
*An. vagus*	1688	11.9	61	0.4	1749	12.4
*An. aconitus*^b^	1286	9.1	210	1.5	1496	10.6
*An. kochi*	912	6.4	289	2.0	1201	8.5
*An. hyrcanus* group	400	2.8	372	2.6	772	5.5
*An. philippinensis*^b^	484	3.4	198	1.4	682	4.8
*An. umbrosus*	246	1.7	32	0.2	278	2.0
*An. barbirostris* s.l.^b^	174	1.2	93	0.7	267	1.9
*An. tessellatus*	117	0.8	42	0.3	159	1.1
*An. jamesii*	25	0.2	25	0.2	50	0.4
*An. dirus* s.l.^a^	10	0.1	33	0.2	43	0.3
*An. splendidus*	22	0.2	11	0.1	33	0.2
*An. jeyporiensis*	24	0.2	1	0.003	25	0.2
*An. argyropus*	20	0.1	0	0	20	0.1
*An. pseudojamesi*	6	0.04	13	0.1	19	0.1
*An. pallidus*^c^	7	0.05	0	0	7	0.05
*An. crawfordi*	0	0	4	0.03	4	0.03
*An. varuna*	1	0.007	1	0.007	2	0.01
*An. aitkenii* group	1	0.007	0	0	1	0.007
*An. barbumbrosus*	1	0.007	0	0	1	0.007
*An. karwari*	1	0.007	0	0	1	0.007
*An. sinensis*	0	0	1	0.007	1	0.007
Total	11,155	78.9	2991	21.1	14,146	100

^a^Primary vector

^b^Secondary vector. A primary vector is a species of *Anopheles* mainly responsible for transmitting malaria in any particular circumstance. A secondary vector is thought to play a lesser role in transmission than the principal vector; capable of maintaining malaria transmission at a reduced level or at particular period of the year

^c^Although *An. pallidus* has been recorded from Laos, it may be a variant form of *An. nivipes* as commented by Reid [55] and it is not present or removed from the lists of mosquitoes in Thailand, Cambodia, Vietnam, Singapore, and Malaysia

The abundance and diversity of the *Anopheles* showed a seasonal trend. As expected, there were more *Anopheles* collected during the rainy season (68%) than during the dry season (32%) and the number of mosquitoes collected varied significantly according to the location. For example, more than 3500 *Anopheles* were collected in Vientiane province alone, against only 150 specimens in Savannakhet, but overall, these results showed that people in rural Lao villages are constantly exposed to malaria vector mosquitoes throughout the year. Furthermore, the study on the mosquito biting preference showed that even if they are highly zoophagic, primary and secondary vectors are biting humans constantly during the night, both indoors and outdoors. Previous studies showed that there was a direct link between close proximity between human and cattle, biting rates and malaria prevalence (i.e. zoopotentiation) [40, 41]. In most of the study sites, cattle and livestock were largely present around the houses from dusk to dawn and the same species were found biting both human and cows but in higher proportion on this latter. Every day before sunset, the cattle owners of the villages bring back their animals to their yard and sometimes under their traditional wooden houses or in a dedicated place in the village, thus increasing the risk for villagers living near these animals to being bitten outdoors. Almost 30% of primary and secondary vectors were collected outdoors before 10:00 PM or after 5:00 AM when people are still outside. This shows the importance of personal protection and other outdoor related control measures, such as zooprophylaxis, to tackle malaria transmission in these remote areas. In the systematic review of Donnelly et al*.* [41], the authors pointed out that zooprophylaxis may be part of Integrated Vector Management (IVM) in areas where the dominant vectors are highly zoophilic and the livestock are kept away from human sleeping quarters. However, their results also showed that when vector preference is opportunist, varied or unknown, there are no evidence to support the use of zooprophylaxis and zoopotentiation could even be increased. In Laos, research regarding this method is clearly needed to validate its usefulness within the large diversity of environments, vectors characteristics and socio-economic factors.

In total, 63% of the vector were collected outdoors which is in adequacy with the results of Chaumeau et al. [42] who estimated that 65% of the potential infective bites are not prevented by bed nets because of outdoor and early biters. Twenty-two percent of the malaria vectors were collected in the villages indoors between 10:00 PM and 5:00 AM when the people are sleeping. Although this represent a relatively low percentage, it highlights the crucial need to provide household with bed nets to protect people during this specific period of the night. Kobayashi et al. [43] already confirmed in the 2000’s, the efficacy of treated bed nets in highly endemic areas of Laos and since the country-wide distribution programmes, implemented by the Ministry of Health, of first ITNs and then LLINs, malaria prevalence has dramatically decreased [44]. Vector control with the use of pyrethroid insecticides in Laos is currently possible as the primary and secondary vectors are still mostly susceptible [9]. However, continuous monitoring and the use of different insecticide family is recommended as resistance is likely to evolve in some parts of the country using high amount of insecticides for pest control. In a recent study, Souris et al. [45] highlighted the space–time distribution of the environmental risk of *Anopheles* presence, potential insecticide emergence, insecticide resistance, and risk of exposure to these threats for the human population in Laos. Their results showed that the probability of insecticide resistance in malaria vectors is greater in the southern part of the country, specifically in Champasak and Attapeu provinces, bordering Cambodia, Thailand and Vietnam. In these areas, malaria incidence is among the highest and the resurgence have been attributed to large-scale population movements (both within Lao and across national borders) as well as forest-related economic activities [5].

Malaria transmission in the Mekong region is currently concentrated in forested and rural areas and along national borders from where the disease is likely to spread to other areas due to the movement of population groups [1, 5, 46]. A part of the transmission is taking place outdoors in the villages as previously mentioned, but the remaining transmission occurs outside of the villages, especially in the forest. In Laos, significant correlations between working and sleeping habits in the forest and malaria incidence were reported [46, 47]. In forested areas, *An. dirus* is thought to be the dominant malaria vector of the southern parts of the country [12, 32, 43, 48]. However, very few specimens were collected in the study (n = 43) and this could be explained by its specific breeding sites usually being located in forested areas nearby the villages [15, 45].

Mosquito collections conducted in forested areas of the Lao–Thailand border at the same period of time, showed that *An. dirus *s.l. was the predominant species biting humans [49] This study showed that during the rainy season, human biting rates of *An. dirus* s.l. (HBR = 0.91) were more than 30 times higher than within village locations (HBR = 0.03). We highly recommend to conduct mosquito collections in the southern forested and remote areas of Laos to better understand the role of *An. dirus* in malaria residual transmission among people working in plantations and forest camps.

This study on malaria parasites infections in the mosquitoes collected could not give us valid indications on the malaria transmission intensity and patterns in our study sites. Indeed, more than 4000 mosquitoes were tested for *Plasmodium* incidence and only two specimens were positives; one *An. aconitus* specimen from Phongsaly province and one *An. minimus *s.s. specimen from Vientiane Province were positive with *P. falciparum*, with a mean sporozoite rate of 0.04%. This confirmed the recent work of [50] where no infected mosquitoes could be found in the Kanchanburi province in Thailand during a malaria outbreak. This also confirms previous studies implemented in the early 2000s in Laos, when malaria transmission was even higher than nowadays [12, 32, 43, 44], sporozoite rates in mosquitoes were very low. These results can be explained by (i) the low number of night collections conducted during the dry and rainy seasons hence limiting the chance to catch *Plasmodium*-positive mosquitoes in low transmission settings, (ii) by the strong zoophagic preferences of both primary and secondary malaria vectors hence limiting the human-vector exposure, and (iii) a low number of malarial parasite carriers due to the bed net coverage and/or and case detection efficacy in the villages.

Currently, research on alternative strategy for vector control is nonexistent in Laos. It is imperative to determine joint research priority axis in Laos and in the GMS with regards to additional vector control tools (VCTs) that could complement insecticide-treated nets (ITNs) and indoor residual spraying (IRS) to achieve malaria elimination [51]. VCTs should take into account the dynamics of the transmission, as well as the ecology of malaria vectors in local settings. For example, veterinary approaches such as the use of insecticide-treated mosquito nets fenced around cattle [52], the use of endectocides by injection in livestock [53] or pyriproxyfen-treated polypropylene sheets and resting boxes for controlling mosquitoes in livestock operations [54] may be interesting strategies to target the zoophilic and exophagic zoophagic malaria vectors (e.g. *An. maculatus*, *An. minimus* and *An. sawadwongporni*). The use of mosquito-proofed housing could be useful to protect people from endophagic mosquitoes such as *An. dirus*, *An. nivipes*, *An. barbirostris*, and *An. philippinensis*. Inthavong et al. [46] clearly showed that households in villages with high malaria incidence were significantly more likely to have an open space on the house surface compared to villages with low incidence.

## Conclusions

Malaria transmission is most likely occurring outside the villages, especially in remote, hilly-forested areas, farms, logging camps, where conventional malaria vector control tools are inefficient. In the context of malaria elimination in Laos, it is a priority to investigate its magnitude by combining entomological, epidemiological, and social surveys. It is also important to provide the most vulnerable population such as migrant and mobile populations (MMPs) with more effective tools for personal protection. This entomological survey also emphasizes the need to continue the distribution of LLINs to prevent people from the substantial part of indoor biting occurring at night and to search for more innovative tools to tackle malaria transmission occurring outdoor.

## Supplementary information

**Additional file 1: Table S1.** Species diversity and abundance of morphologically identified Anopheles mosquitoes collected in Laos during dry and rainy seasons of 2014 and 2015.

**Additional file 2: Table S2.** Sibling species of the Maculatus group determined by PCR and sequencing methods compared to the field morphological identification.

**Additional file 3: Table S3.** Biting times of the Anopheles vectors indoors and outdoors on human.

**Additional file 4: Table S4.** Anopheles species tested for *Plasmodium* sp. infection, Laos.

## Data Availability

The dataset generated during this study is included in this published article and its additional files.

## Abbreviations

AI: Anthropophagic Index
AM: Ante Meridiem (before noon)
AS-PCR: Allele-specific polymerase chain reaction
CBC: Cow bait collections
CBR: Cow biting rate
CMPE: Centre de Malariologie, Parasitologie et Entomologie du Laos
DNA: Deoxyribonucleic acid
dNTP: Deoxynucleoside triphosphates
EnI: Endophagic index
ExI: Exophagic index
GMS: Greater Mekong Subregion
HBR: Human Biting Rate
HLC: Human Landing Catch
IPL: Institut Pasteur du Laos
ITS-2: Internal transcribed spacer 2
Lao PDR: Lao People's Democratic Republic
LLIN: Long-Lasting Insecticide-impregnated mosquito bed Nets
MMP: Mobile and migrant populations
NCGM: National Center for Global Health and Medicine
PCR: Polymerase chain reaction
qPCR: Quantitative polymerase chain reaction
WHO: World Health Organization
ZI: Zoophagic index
