# Supplementary material for: New insights into malaria vector bionomics in Lao PDR: a nationwide entomology survey
Source: Malar J. 2020 Nov 9;19:396. doi: 10.1186/s12936-020-03453-9 (PMC7654023; doi:10.1186/s12936-020-03453-9)
Supplement: Supplementary file 1 — Additional file 1: Table S1. Species diversity and abundance of morphologically identified Anopheles mosquitoes collected in Laos during dry and rainy seasons of 2014 and 2015. [file 12936_2020_3453_MOESM1_ESM.docx]

Additional file 1: Table S1. Species diversity and abundance of morphologically identified *Anopheles* mosquitoes collected in Laos during dry and rainy seasons of 2014 and 2015.

| **Species** | **Vientiane** | **Attapeu** | **Luang Prabang** | **Bokeo** | **Sekong** | **Khammouane** | **Phongsaly** | **Borlikhamxay** | **Saravane** | **Savannakhet** | **Total** | **Percent** |
| --- | --- | --- | --- | --- | --- | --- | --- | --- | --- | --- | --- | --- |
| *An. minimus s.l.* | 963 | 281 | 284 | 125 | 20 | 461 | 249 | 121 | 225 | 18 | **2747** | **19.42** |
| *An. nivipes s.l.* | 612 | 426 | 596 | 52 | 679 | 116 | 0 | 108 | 49 | 70 | **2708** | **19.14** |
| *An. maculatus s.l.* | 18 | 500 | 188 | 42 | 393 | 12 | 645 | 34 | 38 | 9 | **1879** | **13.28** |
| *An. vagus* | 5 | 445 | 185 | 917 | 12 | 151 | 12 | 15 | 6 | 1 | **1749** | **12.36** |
| *An. aconitus* | 1441 | 0 | 8 | 20 | 0 | 11 | 0 | 13 | 0 | 3 | **1496** | **10.58** |
| *An. kochi* | 80 | 86 | 155 | 161 | 168 | 477 | 46 | 24 | 0 | 4 | **1201** | **8.49** |
| *An. hyrcanus s.l.* | 312 | 75 | 239 | 24 | 28 | 2 | 42 | 19 | 22 | 9 | **772** | **5.46** |
| *An. philippinensis* | 131 | 75 | 103 | 0 | 138 | 60 | 1 | 151 | 2 | 21 | **682** | **4.82** |
| *An. umbrosus* | 9 | 6 | 24 | 109 | 14 | 84 | 23 | 5 | 2 | 2 | **278** | **1.97** |
| *An. barbirostris s.l.* | 112 | 12 | 18 | 62 | 8 | 37 | 6 | 9 | 0 | 3 | **267** | **1.89** |
| *An. tessellatus* | 108 | 29 | 4 | 3 | 2 | 4 | 1 | 5 | 3 | 0 | **159** | **1.12** |
| *An. jamesii* | 43 | 1 | 0 | 0 | 4 | 0 | 0 | 1 | 1 | 0 | **50** | **0.35** |
| *An. dirus s.l.* | 0 | 5 | 8 | 0 | 17 | 4 | 3 | 0 | 6 | 0 | **43** | **0.30** |
| *An. splendidus* | 27 | 0 | 0 | 0 | 0 | 0 | 0 | 0 | 0 | 6 | **33** | **0.23** |
| *An. jeyporiensis* | 0 | 25 | 0 | 0 | 0 | 0 | 0 | 0 | 0 | 0 | **25** | **0.18** |
| *An. argyropus* | 14 | 5 | 1 | 0 | 0 | 0 | 0 | 0 | 0 | 0 | **20** | **0.14** |
| *An. pseudojamesi* | 6 | 0 | 12 | 0 | 0 | 0 | 0 | 1 | 0 | 0 | **19** | **0.13** |
| *An. pallidus* | 0 | 0 | 0 | 1 | 3 | 0 | 0 | 3 | 0 | 0 | **7** | **0.05** |
| *An. crawfordi* | 0 | 0 | 0 | 0 | 0 | 0 | 4 | 0 | 0 | 0 | **4** | **0.03** |
| *An. varuna* | 0 | 0 | 1 | 0 | 0 | 0 | 0 | 1 | 0 | 0 | **2** | **0.01** |
| *An. aitkenii* | 0 | 0 | 0 | 0 | 0 | 0 | 1 | 0 | 0 | 0 | **1** | **0.01** |
| *An. barbumbrosus* | 0 | 0 | 0 | 0 | 0 | 0 | 0 | 0 | 1 | 0 | **1** | **0.01** |
| *An. karwari* | 0 | 0 | 0 | 0 | 0 | 0 | 0 | 1 | 0 | 0 | **1** | **0.01** |
| *An. sinensis* | 1 | 0 | 0 | 0 | 0 | 0 | 0 | 0 | 0 | 0 | **1** | **0.01** |
| *An. sawadwongporni* | 1 | 0 | 0 | 0 | 0 | 0 | 0 | 0 | 0 | 0 | **1** | **0.01** |
| **Total** | **3883** | **1971** | **1826** | **1516** | **1486** | **1419** | **1033** | **511** | **355** | **146** | **14146** | **100** |
| **Percent** | **27.46** | **13.94** | **12.91** | **10.72** | **10.51** | **10.03** | **7.30** | **3.61** | **2.51** | **1.03** |  |  |
