# Supplementary material for: New insights into malaria vector bionomics in Lao PDR: a nationwide entomology survey
Source: Malar J. 2020 Nov 9;19:396. doi: 10.1186/s12936-020-03453-9 (PMC7654023; doi:10.1186/s12936-020-03453-9)
Supplement: Supplementary file 2 — Additional file 2: Table S2. Sibling species of the Maculatus group determined by PCR and sequencing methods compared to the field morphological identification. [file 12936_2020_3453_MOESM2_ESM.docx]

**Additional file 2: Table S2.**

**A. Sibling species of the Maculatus group determined by PCR and sequencing methods compared to the field morphological identification.**

**Note:** Negative = extraction or sequencing failed; Field misidentification of mosquitoes in different *Anopheles* groups resulted in the numbers of field-identified mosquitoes being different to the numbers of mosquitoes identified by PCR/sequencing.

**B. Sibling species of the Funestus group determined by PCR and sequencing methods compared to the field morphological identification.**

**Note:** Negative = extraction or sequencing failed; Field misidentification of mosquitoes in different *Anopheles* groups resulted in the numbers of field-identified mosquitoes being different to the numbers of mosquitoes identified by PCR/sequencing.

**C. Sibling species of the Leucosphyrus group determined by PCR and sequencing methods compared to the field morphological identification.**
