# Supplementary material for: New insights into malaria vector bionomics in Lao PDR: a nationwide entomology survey
Source: Malar J. 2020 Nov 9;19:396. doi: 10.1186/s12936-020-03453-9 (PMC7654023; doi:10.1186/s12936-020-03453-9)
Supplement: Supplementary file 3 — Additional file 3: Table S3. Biting times of the Anopheles vectors indoors and outdoors on human. [file 12936_2020_3453_MOESM3_ESM.docx]

Additional file 3: Table S3: Biting times of the *Anopheles* vectors indoors and outdoors on human
