# Supplementary material for: New insights into malaria vector bionomics in Lao PDR: a nationwide entomology survey
Source: Malar J. 2020 Nov 9;19:396. doi: 10.1186/s12936-020-03453-9 (PMC7654023; doi:10.1186/s12936-020-03453-9)
Supplement: Supplementary file 4 — Additional file 4: Table S4. Anopheles species tested for Plasmodium sp. infection, Laos. [file 12936_2020_3453_MOESM4_ESM.docx]

**Additional file 4: Table S4. *Anopheles* species tested for *Plasmodium* sp. infection, Laos.**

| **Species** | **N tested** | **Positive** |
| --- | --- | --- |
| *An. aconitus* | 1538 | 1 |
| *An. annularis s.l.* | 1 | 0 |
| *An. baimaii* | 5 | 0 |
| *An. dirus* | 42 | 0 |
| *An. dravidicus* | 105 | 0 |
| *An. harrisoni* | 30 | 0 |
| *An. karwari* | 3 | 0 |
| *An. kochi* | 9 | 0 |
| *An. maculatus s.s.* | 454 | 0 |
| *An. minimus s.s.* | 959 | 1 |
| *An. nemophilous* | 1 | 0 |
| *An. nivipes s.l.* | 8 | 0 |
| *An. pampanai* | 151 | 0 |
| *An. philippinensis* | 4 | 0 |
| *An. pseudowillmori* | 91 | 0 |
| *An. rampae* | 458 | 0 |
| *An. sawadwongporni* | 198 | 0 |
| *An. sinensis* | 1 | 0 |
| *An. splendidus* | 1 | 0 |
| *An. subpictus* | 1 | 0 |
| *An. tessellatus* | 7 | 0 |
| *An. vagus* | 73 | 0 |
| *An. willmori* | 52 | 0 |
| **TOTAL** | **4192** | **2** |
